# The role of surgical resection before palliative chemotherapy in advanced gastric cancer

**DOI:** 10.1038/s41598-019-39432-7

**Published:** 2019-03-11

**Authors:** Yong Won Choi, Mi Sun Ahn, Geum Sook Jeong, Hyun Woo Lee, Seong Hyun Jeong, Seok Yun Kang, Joon Seong Park, Jin-Hyuk Choi, Sang-Yong Son, Hoon Hur, Sang-Uk Han, Seung Soo Sheen

**Affiliations:** 1 Department of Hematology-Oncology, Ajou University School of Medicine, Suwon, Korea; 2 Department of Surgery, Ajou University School of Medicine, Suwon, Korea; 3 Department of Pulmonary and Critical Care Medicine, Ajou University School of Medicine, Suwon, Korea

## Abstract

The role of palliative surgical resection in recurrent or metastatic gastric cancer is still controversial. A retrospective review was conducted on 689 patients who received palliative chemotherapy for recurrent (n = 307) or primary metastatic (n = 382) gastric cancer. Among 131 patients (89 primary metastatic and 42 recurrent) with surgical resection before chemotherpay, 75 underwent gastrectomy, 42 metastasectomy, and 14 gastrectomy with metastasectomy. The median overall survival (OS) of patients who underwent surgical resection was significantly longer than that of patients who received chemotherapy alone (18 vs. 9 months, *p* < 0.0001). The OS benefit of surgical resection was consistent across subgroups. In multivariate analysis, surgical resection was independently associated with favorable OS (hazard ratio = 0.42, *p* < 0.0001). Moreover, patients with surgical resection showed favorable OS both in univariate (*p* < 0.0001) and multivariate (*p* < 0.0001) analysis even after pro*p*ensity score matching. In addition, the median OS of patients who underwent gross complete resection (n = 54) was significantly longer than that of patients who underwent incomplete resection (n = 77) (30 vs. 15 months, *p* = 0.002). The present study suggests that judicious use of surgical resection before chemotherapy in recurrent or metastatic gastric cancer patients may result in a favorable outcome, especially when complete resection is achievable.

## Introduction

Gastric cancer is the most common type of cancer in Korea and the third leading cause of cancer-related mortality in the world^1,2^. While palliative chemotherapy is the standard of care for patients with recurrent or primary metastatic gastric cancer (RPMGC), surgical resection (metastasectomy, palliative gastrectomy with or without metastasectomy) is often performed for patients with potentially resectable lesions in practice. In addition, palliative gastrectomy is generally indicated in cases of potentially life-threatening problems due to obstruction, perforation, or bleeding^3,4^.

However, the role of palliative surgical resection is still controversial in general treatment practice for RPMGC. Previous retrospective studies^4–13^, meta-analysis^14–17^, and population based analysis^18–22^ have suggested that the addition of surgical resection to chemotherapy may have a survival benefit in RPMGC. However, the only randomized phase III trial, the REGATTA study^3^, failed to prove a survival benefit of gastrectomy followed by chemotherapy compared with chemotherapy alone in primary metastatic gastric cancer patients. Consequently, it is an important clinical issue to determine whether surgical resection before palliative chemotherapy provides improved outcome compared to chemotherapy alone and to identify patients who may benefit from surgical resection.

Therefore, this study retrospectively compared the outcomes between RPMGC patients who underwent palliative resection before first-line chemotherapy and those who received chemotherapy alone, while analyzing patients characteristics that may be associated with prognosis.

## Patients and Methods

### Study population

This study retrospectively identified all RPMGC patients who started first-line palliative chemotherapy between January 2004 and December 2014 at Ajou University Hospital, Suwon, Korea. Histologically confirmed patients with RPMGC were eligible. In cases of primary metastatic disease, American Joint Committee on Cancer (AJCC) stage IV^23^ gastric cancer with distant metastasis or gross residual tumor after surgical resection were included. Among stage IV patients, those with distant abdominal lymph node metastasis (e.g. retropancreatic or mesenteric) or tumor cells in peritoneal cytology only were excluded if complete resection of the primary tumor and regional lymph node dissection without gross residual disease were performed according to the General Rules for the Gastric Cancer Study in Surgery and Pathology of the Japanese Research Society for Gastric Cancer^24^. Patients who had intiated first-line chemotherapy at other hospitals during this period and received further first-line therapy with the same regimen at our institution were included.

All procedures in the study involving human participants were carried out in accordance with the ethical standards of the institutional and/or national ethical committee and with the 1964 Helsinki Declaration and its later amendments or comparable ethical standards. The protocol was reviewed and approved by the Institutional Review Board (IRB) of Ajou University Hospital (IRB approval no. AJIRB-MED-MDB-16-022). The informed consent of this study was waived by the IRB, given its retrospective nature using anonymized data. A study about palliative chemotherapy for RPMGC patients, which included the majority of patients in the current study cohort, was previously reported^25^.

### Clinical review

The medical records of patients were reviewed retrospectively. Data on patients were collected, including patients characteristics (gender, age, performance status (PS) based on the Eastern Cooperative Oncology Group (ECOG) performance scale, histology, disease status at diagnosis, peritoneal and liver metastasis, surgical resection before start of first-line chemotherapy, chemotherapy regimens including lines) and survival information. For histologic subclassification, pathologic information on primary tumor of stomach was used in both primary metastatic and recurrent disease. In patients with local recurrence, histology was classified according to the pathology report on the recurrent stomach lesion if available.

### Statistical analysis

Overall survival (OS) was calculated using the Kaplan–Meier method. OS was defined as the time from the starting day of the first-line chemotherapy to death. Data on survivors was censored as of the last follow-up. Differences between the survival curves were analyzed by the log-rank test. Fisher’s exact test was used to compare the different groups for categorical variables. The Cox proportional hazards regression model was used to determine the joint effects of several variables on survival and to assess interactions between treatment and subgroup in subgroup analyses. Factors with *p* values < 0.1 in univariate analysis were included in the Cox proportional hazards regression model. All statistical analyses were two-sided and performed with SPSS version 23.0 for Windows.

We used propensity score matching (PSM), the 1:1 nearest neighbor matching, to minimize the selection bias by adjusting variables that may affect the survival of patients. SPSS version 23.0 for Windows was also used for PSM.

## Results

### Patient characteristics

Between January 2004 and December 2014, 685 patients initiated first-line palliative chemotherapy for PRMGC at our institution. In addition, four patients who had started first-line chemotherapy at other hospitals during this period received further first-line therapy with the same regimen at our institution. Therefore, a total of 689 patients were included for analysis. The patients’ clinicopathological characteristics are summarized in Table 1. Of the 689 patients, 477 (69.2%) were male, 127 (18.4%) were ≥70 years, 611 (88.7%) were in ECOG PS 0 or 1, and 187 (27.1%) had poorly differentiated subtype. A total of 310 (45.0%) and 157 (22.8%) patients had peritoneal and liver metastasis, respectively (both: 33 patients). Among patients with primary metastatic disease, all patients were in AJCC stage IV except for two stage III patients with gross residual disease after resection. Of the 307 (44.6%) recurrent disease patients, 277 had undergone adjuvant chemotherapy.

**Table 1:** Patients characteristics at the initiation of first-line chemotherapy.

Characteristics	Before propensity score matching			*p*	After propensity score matching			*p*
	Total *N* (%)	Surgical resection *N* (%)			Total *N* (%)	Surgical resection *N* (%)		
		Yes	No			Yes	No	
Gender								
Male	477 (69.2)	84 (64.1)	393 (70.4)	0.172	168 (64.1)	84 (64.1)	84 (64.1)	1.000
Female	212 (30.8)	47 (35.9)	165 (29.6)		94 (35.9)	47 (35.9)	47 (35.9)	
Age (years)								
<70	562 (81.6)	117 (89.3)	445 (79.7)	0.012	234 (89.3)	117 (89.3)	117 (89.3)	1.000
≥70	127 (18.4)	14 (10.7)	113 (20.3)		28 (10.7)	14 (10.7)	14 (10.7)	
PS (ECOG)								
0, 1	611 (88.7)	124 (94.7)	487 (87.3)	0.014	251 (95.8)	124 (94.7)	127 (96.9)	0.540
2, 3	78 (11.3)^*^	7 (5.3)	71 (12.7)		11 (4.2)	7 (5.3)	4 (3.1)	
Disease status								
Primary metastatic	382 (55.4)	89 (67.9)	293 (52.5)	0.002	179 (68.3)	89 (67.9)	90 (68.7)	1.000
Recurrent	307 (44.6)	42 (32.1)	265 (47.5)		83 (31.7)	42 (32.1)	41 (31.3)	
Tumor differentiation (WHO)								
Well, moderate	169 (24.5)	19 (14.5)	150 (26.9)	0.003	36 (13.7)	19 (14.5)	17 (13.0)	0.984
Poor	187 (27.1)	31 (23.7)	156 (28.0)		63 (24.0)	31 (23.7)	32 (24.4)	
Signet ring cell	169 (24.5)	41 (31.3)	128 (22.9)		84 (32.1)	41 (31.3)	43 (32.8)	
Combined, others	164 (23.8)	40 (30.5)	124 (22.2)		79 (30.2)	40 (30.5)	39 (29.8)	
Peritoneal metastasis								
No	379 (55.0)	73 (55.7)	306 (54.8)	0.922	142 (54.2)	73 (55.7)	69 (52.7)	0.710
Yes	310 (45.0)	58 (44.3)	252 (45.2)		120 (45.8)	58 (44.3)	62 (47.3)	
Liver metastasis								
No	532 (77.2)	113 (86.3)	419 (75.1)	0.005	225 (85.9)	113 (86.3)	112 (85.5)	1.000
Yes	157 (22.8)	18 (13.7)	139 (24.9)		37 (14.1)	18 (13.7)	19 (14.5)	
1st line CTx								
Single	170 (24.7)	27 (20.6)	143 (25.6)	0.261	44 (16.8)	27 (20.6)	17 (13.0)	0.136
Combination	519 (75.3)	104 (79.4)	415 (74.4)		218 (83.2)	104 (79.4)	114 (87.0)	

N: number, PS: performance status, ECOG: Eastern Cooperative Oncology Group, CTx: chemotherapy. ^*^PS 3: 2 patients.

Palliative surgical resection before first-line chemotherapy was performed in 131 patients (19.0%). A total of 75 (57.3%), 42 (32.1%) and 14 (10.7%) patients underwent gastrectomy, metastasectomy, and both, respectively (Table 2). In almost all patients, surgical resection was planned by the decision of surgeons after preoperative staging work-up including abdominopelvic CT scan. Gastrectomy with resection of regional lymph nodes or a small number of peritoneal metastatic nodules was categorized as gastrectomy (Table 2). Gross complete resection (GCR), defined as the complete removal of distant metastatic lesion(s) and/or primary tumor without gross residual disease as well as any metastatic lesion in other sites, was performed in 54 patients (41.2%) (primary metastatic: 23 patients, recurrent disease: 31 patients). The reasons for performing palliative surgical resection in primary metastatic disease are: (1) stage IV disease with distant metastasis or presence of gross residual tumor after surgery, despite expectation of curative resection in preoperative studies (56 patients); (2) potentially life-threatening problems such as bleeding, obstruction, or perforation (14 patients); (3) completely resectable both primary and distant metastatic lesion(s) in preoperative study (13 patients); (4) enrollment in the REGATTA trial^3^ (3 patients); (5) uncategorized (3 patients). In recurrent disease, the reasons for surgical resection were completely resectable lesion(s) on preoperative evaluation (36 patients) and the palliation of major symptoms (6 patients). The type of palliative surgical resection according to disease status and completeness of resection including the sites of metastasectomy is summarized in Table 2. The most frequent site of metastasectomy was ovary (20 patients including 2 patients with metastasis to uterus), followed by liver (14 patients), colon (7 patients), small intestine (3 patients), distant lymph nodes (2 patients), spleen (2 patients), others (6 patients), and multiple (2 patients).

**Table 2:** Type of palliative surgical resection according to disease status and completeness of resection including the sites of metastasectomy.

	Total *N* (%)	Disease status	
		Primary metastatic	Recurrent
Surgical resection	131	89	42
Gastrectomy	75 (57.3)	73 (82.0)	2 (4.8)
Gross complete resection	15 (20.0)	13 (17.8)^+^	2 (100)
Incomplete resection	60 (80.0)	60 (82.2)	0 (0.0)
Metastasectomy	42 (32.1)	4 (4.5)	38 (90.5)
Gross complete resection	27 (64.3)	0 (0.0)	27 (71.1)
Ovary	10	0	10
Liver	8	0	8
Colon	2	0	2
Small intestine	1	0	1
Distant lymph nodes	1	0	1
Others^*^	5	0	5 (18.5)
Incomplete resection	15 (35.7)	4 (100.0)	11 (28.9)
Ovary	5	1	4
Colon	4	1	3
Small intestine	2	1	1
Distant lymph nodes	1	0	1
Others^*^	3	1	2
Both	14 (10.7)	12 (13.5)	2 (4.8)
Gross complete resection	12 (85.7)	10 (83.3)	2 (100)
Ovary	3	3	0
Liver	6	5	1
Colon	1	1	0
Spleen	2	1	1
Incomplete resection	2 (14.3)	2 (16.7)	0 (0.0)
Ovary	2	2	0

N: number. *Including multiple sites. ^+^All patients underwent gastrectomy with resection of small number of peritoneal metastatic nodules.

First-line chemotherapy was combination for 519 patients (75.3%) and single agent for 170 patients (24.7%). Combination therapy included: 5-FU/leucovorin/oxaliplatin (355 patients); S1/cisplatin (75); capecitabine/oxaliplatin (22); capecitabine or 5-FU/cisplatin/trastuzumab (9); and others (58). Single agent therapy included S1 (150 patients) and others (20). While 354 patients (51.4%) received second- or further-line therapy, 16 patients were transferred to other hospitals during or after first-line therapy without further treatment information. Among 293 patients with primary metastatic disease without surgical resection before chemotherapy, twenty-one patients underwent palliative resection (gastrectomy: 17, metastasectomy: 3, both:1) after initiation of chemotherapy. The reasons for surgical resection were significant response of tumor lesions after chemotherapy (12 patients), bleeding or obstruction (6 patients), and symptom palliation (3 patients). Despite surgical resection, these patients were categorized as chemotherapy alone group in all analysis.

In terms of baseline characteristics, patients with surgical resection before chemotherapy were associated with a high proportion of younger age (<70 years) (*p* = 0.012), good PS (*p* = 0.014), primary metastatic disease (*p* = 0.002), signet ring cell histology (*p* = 0.003), and absence of liver metastasis (*p* = 0.005) in comparison with those who received chemotherapy alone (Table 1). Second- or further-line chemotherapy was more frequently performed in patients with surgical resection compared to those with chemotherapy alone (64.1% vs. 48.4%, p = 0.001). For PSM, clinicopathological characteristics at the start of first-line chemotherapy were used as covariates, which were well balanced after 1:1 PSM (Table 1). In terms of completeness of surgical resection, the baseline characteristics of patients with incomplete resection showed similar findings except age as in entire patients who underwent surgical resection, in comparison with those who underwent chemotherapy alone (Table 3). On the other hand, among patients who received palliative surgical resection, GCR was more frequently performed in patients with younger age (<70 years) (*p* = 0.008), recurrent disease (*p* < 0.0001), and liver metastasis (*p* = 0.001) (Table 3). The most frequent reason for incomplete resection was multiple metastatic lesions such as peritoneal carcinomatosis (50 patients), followed by unresectable gross residual tumors (21 patients), enrollment in the REGATTA trial (3 patients), and operation due to life threatening emergency such as perforation (3 patients).

**Table 3:** Patients characteristics at the initiation of first-line chemotherapy according to the completeness of surgical resection. N: number, PS: performance status, ECOG: Eastern Cooperative Oncology Group, CTx: chemotherapy.

Characteristics	No surgical resection *N* (%)^1^	Gross complete resection *N* (%)		*P*	*P*	*P*
		Yes^2^	No^3^	*(1 vs 2)*	*(1 vs 3)*	*(2 vs 3)*
Gender						
Male	393 (70.4)	31 (57.4)	53 (68.8)	0.063	0.791	0.199
Female	165 (29.6)	23 (42.6)	24 (31.2)			
Age (years)						
<70	445 (79.7)	53 (98.1)	64 (83.1)	<0.0001	0.545	0.008
≥70	113 (20.3)	1 (1.9)	13 (16.9)			
PS (ECOG)						
0,1	487 (87.3)	51 (94.4)	73 (94.8)	0.186	0.059	1.000
2	71 (12.7)	3 (5.6)	4 (5.2)			
Disease status						
Primary metastatic	293 (52.5)	23 (42.6)	66 (85.7)	0.199	<0.0001	<0.0001
Recurrent	265 (47.5)	31 (57.4)	11 (14.3)			
Tumor differentiation (WHO)						
Well, moderate	150 (26.9)	10 (18.5)	9 (11.7)	0.086	0.006	0.239
Poor	156 (28.0)	11 (20.4)	20 (26.0)			
Signet ring cell	128 (22.9)	13 (24.1)	28 (36.4)			
Combined, other	124 (22.2)	20 (37.0)	20 (26.0)			
Peritoneal metastasis						
No	306 (54.8)	37 (68.5)	36 (46.8)	0.062	0.223	0.020
Yes	252 (45.2)	17 (31.5)	41 (53.2)			
Liver metastasis						
No	419 (75.1)	40 (74.1)	73 (94.8)	0.87	<0.0001	0.001
Yes	139 (24.9)	14 (25.9)	4 (5.2)			
1st line CTx						
Single	143 (25.6)	11 (20.4)	16 (20.8)	0.511	0.402	1.000
Combination	415 (74.4)	43 (79.6)	61 (79.2)			

### Overall survival

**Figure 1:**
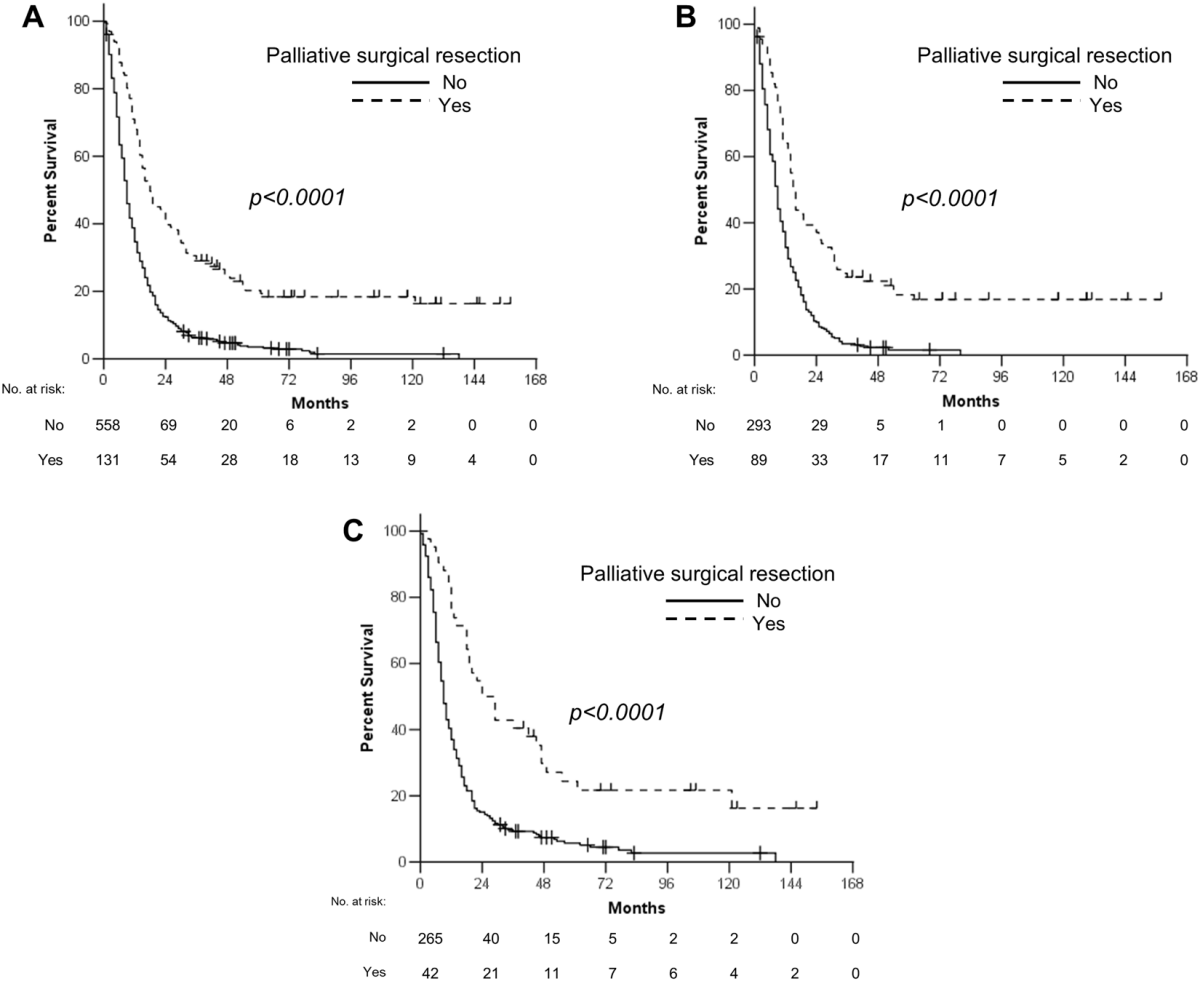
Overall survival according to therapeutic modality for all patients (**A**) and patients with primary metastatic (**B**) or recurrent disease (**C**).

**Figure 2:**
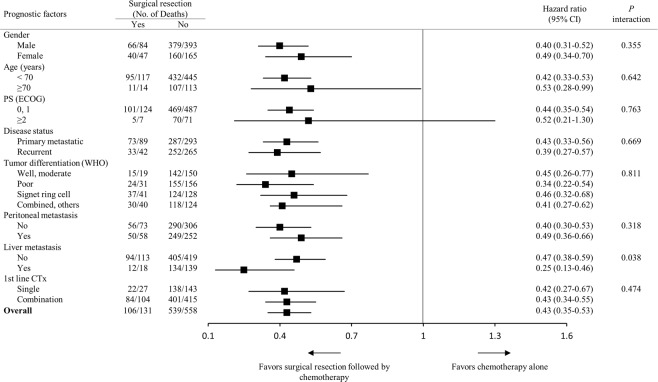
Forest plot for subgroup analyses of overall survival: the effect of surgical resection according to baseline characteristics. CI: confidence interval, PS: performance status; ECOG: Eastern Cooperative Oncology Group, CTx: chemotherapy.

The median follow-up duration for surviving patients was 70 months (31–158 months). Only one patient was lost to follow-up for survival status at one month after the initiation of first-line chemotherapy. Forty-three patients (6.2%) were still alive at the last follow-up time. The median OS of all patients after the initiation of first-line therapy was 10 months. The median OS of patients who underwent palliative surgical resection was longer than that of patients who received palliative chemotherapy alone (18 vs. 9 months, *p* < 0.0001, Fig. 1A). The OS benefit of surgical resection was consistent across subgroups in terms of baseline characteristics including disease status (Figs 1B,C and 2). When the sites of metastasectomy were categorized as four groups (ovary, liver, intestine, and others including multiple) in 56 patients who underwent metastasectomy with or without palliative gastrectomy, there was no statistically significant difference in OS in terms of the site of metastasectomy (*p* = 0.487).

**Figure 3:**
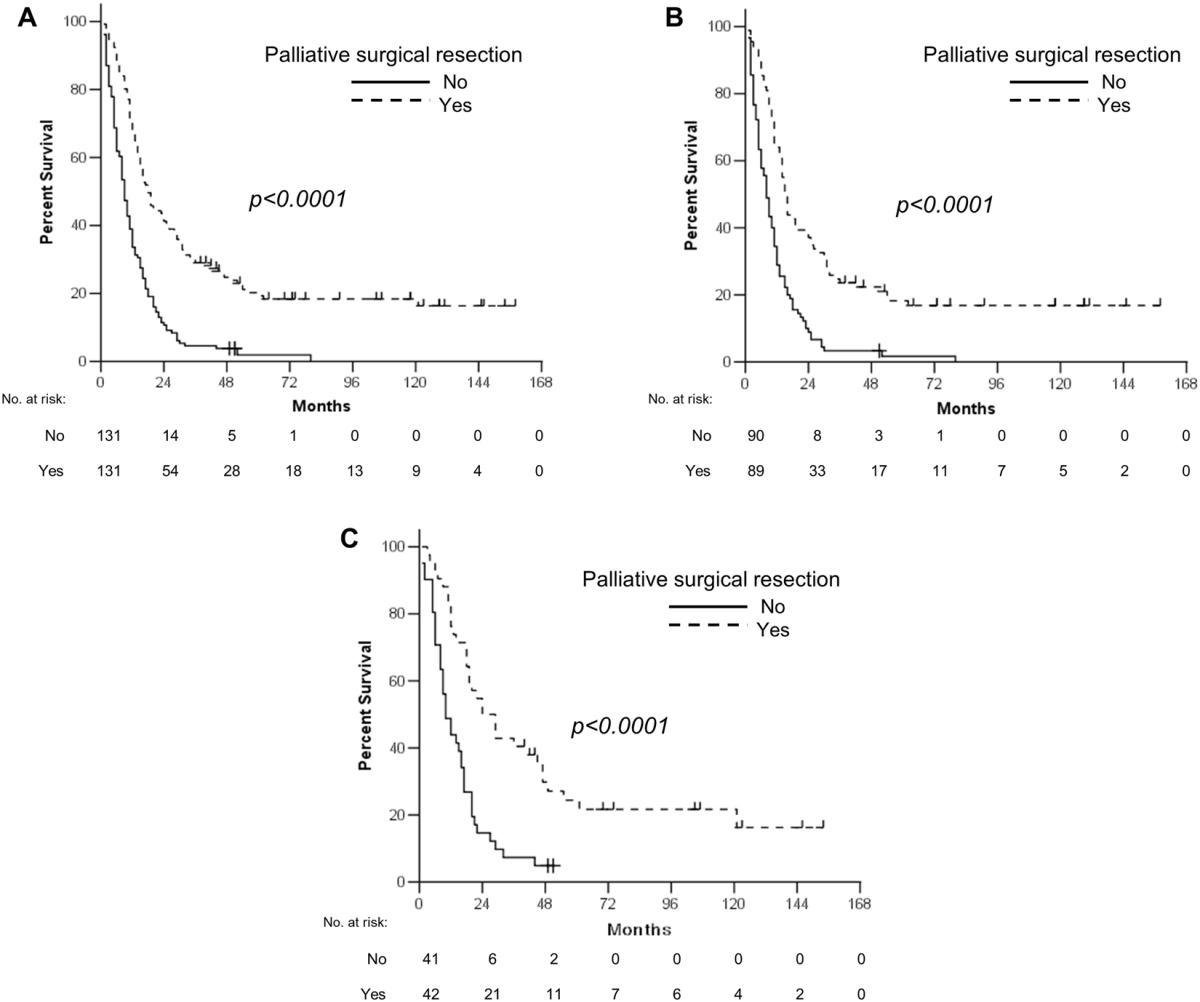
Overall survival according to therapeutic modality for all patients (**A**) and patients with primary metastatic (**B**) or recurrent disease (**C**) after propensity score matching.

In univariate analysis, patients who underwent first-line combination chemotherapy (*p* = 0.001) and second- or further-line therapy (*p* < 0.0001) showed longer median OS, as in surgical resection. Old age (≥70 years) (*p* = 0.038), ECOG PS 2 or 3 (*p* < 0.0001), signet ring cell histology (*p* = 0.029), and presence of peritoneal metastasis (*p* < 0.0001) were associated with poor OS (Table 4). The multivariate analysis revealed that surgical resection was independently associated with favorable OS (hazard ratio = 0.42, *p* < 0.0001), along with second- or further-line therapy (*p* < 0.0001), whereas ECOG PS 2 or 3 (*p* = 0.003), signet ring cell and poorly differentiated histology (*p* < 0.0001 and *p* = 0.047, respectively) and peritoneal metastasis (*p* = 0.012) were independent prognostic factors of poor OS (Table 4). Even after PSM, palliative surgical resection correlated with longer OS (18 vs 9 months, *p* < 0.0001, Fig. 3A), regardless of disease status (Fig. 3B,C), compared with chemotherapy alone in univariate analysis, with independent favorable prognostic significance (hazard ratio = 0.38, *p* < 0.0001) in multivariate analysis (Table 4).

**Table 4:** Univariate and multivariate analysis of overall survival for patients from the start of first-line chemotherapy.

Prognostic factors	Before propensity score matching					After propensity score matching				
	Univariate		Multivariate			Univariate		Multivariate		
	MS	*p*	HR	95% CI	*p*	MS	*p*	HR	95% CI	*p*
Gender										
Male	10	0.857				12	0.855			
Female	10					13				
Age (years)										
< 70	10	0.038	1		0.496	13	0.515			
≥70	9		0.92	0.73–1.16		12				
PS (ECOG)										
0, 1	11	<0.0001	1			13	0.377			
≥2	5		1.49	1.14–1.94	0.003	7				
Disease status										
Primary metastatic	10	0.133				11	0.029	1	0.56–1.03	0.073
Recurrent	10					17		0.76		
Tumor differentiation (WHO)										
Well, moderate	12	0.029	1			17	0.027	1	0.85–2.03	0.225
Poor	10		1.25	1.00–1.56	0.047	15		1.31		
Signet ring cell	8		1.58	1.25–2.00	<0.0001	8		1.90	1.23–2.94	0.004
Combined, others	11		1.19	0.95–1.50	0.14	13		1.30	0.85–1.98	0.233
Peritoneal metastasis										
No	11	<0.0001	1			14	0.004	1	0.87–1.60	0.283
Yes	9		1.23	1.05–1.45	0.012	11		1.18		
Liver metastasis										
No	10	0.632				12	0.089	1	0.66–1.52	0.996
Yes	9					14		1.00		
Surgical resection										
No	9	<0.0001	1			9	<0.0001	1	0.29–0.49	<0.0001
Yes	18		0.42	0.34–0.52	<0.0001	18		0.38		
1st line CTx										
Single	8	0.001	1			12	0.861			
Combination	11		0.85	0.68–1.05	0.138	13				
Number of CTx cycles										
1st line CTx	6	<0.0001	1			9	0.187			
≥2nd line CTx	14		0.66	0.56–0.77	<0.0001	15				

MS: median survival (months), HR: hazard ration, CI: confidence interval, PS: performance status, ECOG: Eastern Cooperative Oncology Group, CTx: chemotherapy.

**Figure 4:**
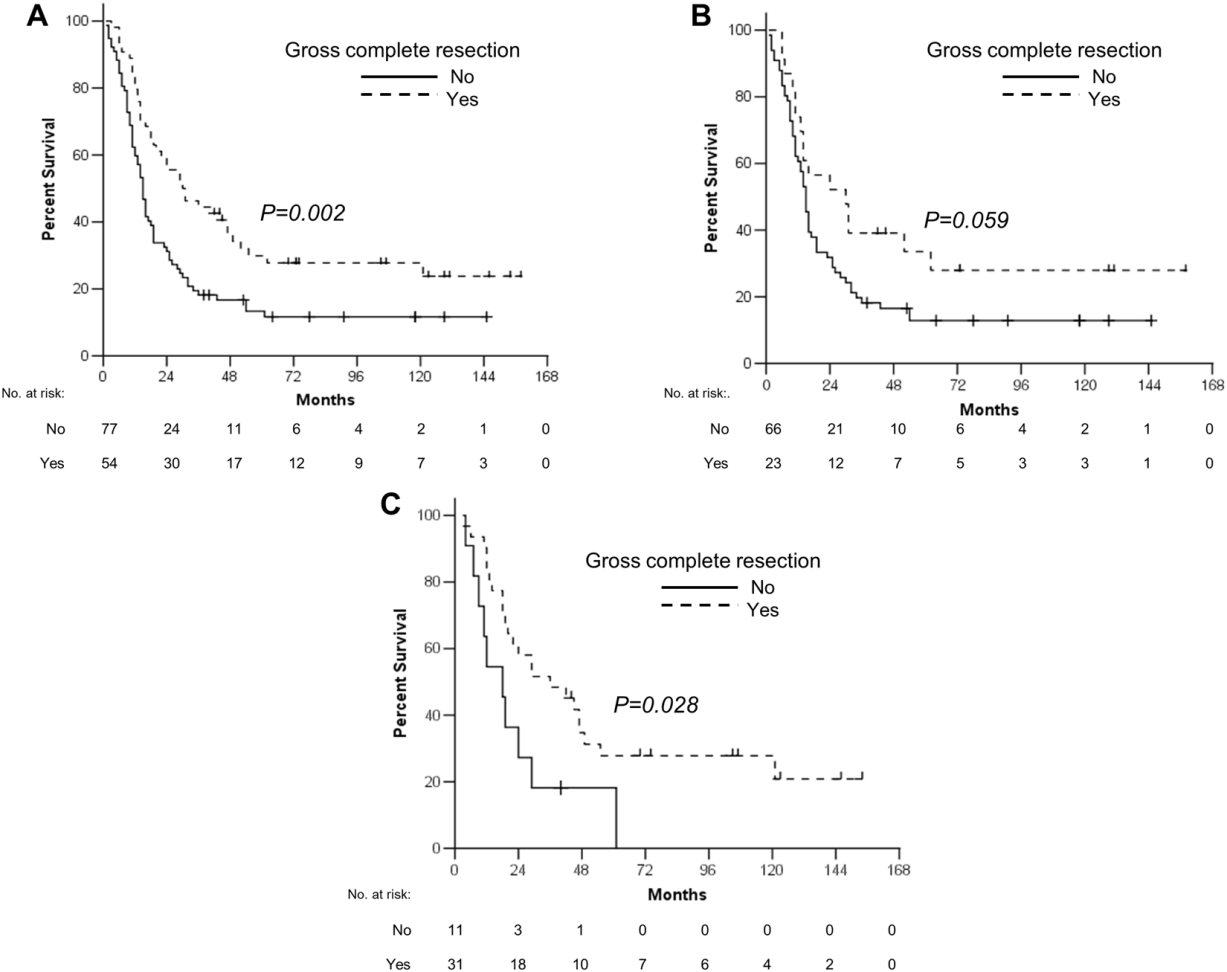
Overall survival according to the completeness of surgical resection for all patients (**A**) and patients with primary metastatic (**B**) or recurrent disease (**C**).

Among patients who underwent surgical resection, the median OS of patients with GCR was significantly longer than that of patients with incomplete resection (30 vs. 15 months, *p* = 0.002, Fig. 4A). On the other hand, even the patients with incomplete resection showed better median OS compared to those with chemotherapy alone (15 vs. 9 months, <0.0001), In patients with primary metastatic disease who underwent incomplete palliative gastrectomy (60 patients), the median OS was 15 months. The OS benefit of GCR in recurrent disease (36 vs. 18 months, *p* = 0.028) was more significant than that of GCR in primary metastatic disease (30 vs. 15 months, *p* = 0.059) (Fig. 4B,C).

## Discussion

In the present study, about 20% of RPMGC patients underwent palliative surgical resection before chemotherapy. The patient cohort consisted of those who had started first-line chemotherapy at our institution since 2004, when third-generation agents such as oxaliplatin and S1^26–28^ was approved for reimbursement in palliative chemotherapy of gastric cancer from the Korean National Health Insurance System. Palliative surgical resection was more frequently performed in patients with relatively young age and good PS (ECOG 0 or 1), as expected in general practice.

The median OS of patients who underwent palliative surgical resection before the initiation of first-line chemotherapy was significantly longer than that of patients who received chemotherapy alone. Moreover, palliative surgical resection itself was an independent favorable prognostic factor in multivariate analysis. The OS benefit of surgical resection was consistently demonstrated across subgroups in terms of various baseline characteristics (e.g. ECOG PS, primary metastatic vs. recurrent disease, single vs. combination first-line chemotherapy). The median OS of 18 months in patients with surgical resection appears encouraging, considering previously reported median OS of RPMGC patients who received palliative chemotherapy^11,26–30^. Nonetheless, randomized trials are necessary to prove whether this OS benefit was caused by the effect of palliative surgical resection itself or the patients’ favorable baseline characteristics, although the beneficial effect of surgical resection was consistent even after PSM including age and PS.

Although many studies^4–17^ have suggested the OS benefit of surgical resection such as palliative gastrectomy or metastasectomy, especially for liver metastasis, in RPMGC, the role of palliative resection is still controversial due to high probability of selection bias in most studies^3^ and some reports showing lack of definite benefit of surgery^31–33^. Furthermore, the results of most previous retrospective studies^4–13^ and meta-analysis^14–17^ were derived from heterogeneous patient populations, including those who had not received chemotherapy as well as those who had undergone it.

Only a few studies have compared the outcomes between surgical resection followed by palliative chemotherapy and chemotherapy alone using cohorts in which all patients underwent chemotherapy. For primary metastatic gastric cancer, two single center^8,12^ and one national cancer database^18^ retrospective studies demonstrated the survival benefit of surgical resection. In addition, one study investigated the role of surgical resection in both primary metastatic and recurrent disease, only in cases of Krukenberg tumor, reporting the survival benefit of metastasectomy^13^. To our knowledge, the present study is the first report to show the OS benefit of surgical resection by analyzing the entire cohort of advanced gastric cancer patients treated with palliative chemotherapy including both primary metastatic and recurrent disease.

The benefit of palliative surgical resection in metastatic cancer has the following biologic basis in general. The chances of disease progression can be diminished theoretically by removing gross tumor, which may be a potential ongoing source of new metastases^3,34^. Decreasing tumor burden by surgical resection could also improve the efficacy of chemotherapy by increasing toleration with relief of tumor-relative symptoms and by reducing the appearance of chemo-resistant clones^3,4,34,35^. In addition, some anti-tumor effects could be anticipated after removing potential source of immunosuppressive cytokines or angiogenic growth factors, such as vascular endothelial growth factor^3,4,34,36,37^.

Despite theoretical advantage of palliative surgical resection with many reports showing survival benefit, as mentioned, a major limitation of retrospective studies and meta-analysis, including the present study, is the inherent selection bias, according to which relatively healthy patients with limited tumor burden have more chance of being selected for aggressive surgical procedures^31^. Therefore, a prospective randomized trial is essential to avoid such selection bias. To date, the REGATTA study conducted in Korea and Japan is the only randomized phase III trial designed to assess the survival benefit of gastrectomy followed by chemotherapy, compared with chemotherapy alone, in primary metastatic gastric cancer, without demonstrating the benefit of gastrectomy^3^. This trial selected only patients with limited metastatic lesions, while resection of distant metastatic tumor or lymph nodes, except perigastric lymph nodes, was not allowed^3^, unlike the present study including patients with resection of metastatic lesions. The median OS of 15 months in primary metastatic disease patients with incomplete palliative gastrectomy in the current study was almost comparable to that of gastrectomy plus chemotherapy and chemotherapy alone arms (14.3 and 16.6 months, respectively) in the REGATTA trial^3^ also including only patients with incomplete resection. This result appears to be clinically meaningful and in line with the conclusion of the REGATTA trial^3^, pointing away from the routine use of palliative gastrectomy in patients without potentially life threatening problems.

The REGATTA trial suggested that incomplete palliative gastrectomy should not be considered in routine practice for primary metastatic gastric cancer patients^3^. On the other hand, in a retrospective analysis for primary metastatic disease, Kim *et al*. showed that the median OS of the patients who underwent GCR of primary and metastatic sites was significantly longer than that of patients who underwent only debulking gastrectomy (28.0 vs 15.5 months)^8^. Similarly, in the present study, the median OS of patients with GCR was significantly longer than that of patients with incomplete resection with quite favorable outcome (median OS: 30 months). Therefore, considering low surgical morbidity and mortality in experienced centers^3,8,15,38^, these results^8^ suggest that surgical resection could be recommended after careful selection.

In primary metastatic disease, patients with major symptoms such as severe bleeding, obstruction unmanageable by non-surgical intervention, or perforation should be considered for palliative gastrectomy. In addition, considering the encouraging OS demonstrated in the present study and the previous report^8^, patients with completely resectable tumors, including metastatic sites, can be potential candidates for surgical resection after a thorough preoperative staging work-up including PET-CT.

On the other hand, the role of surgical resection could be more obvious in patients with recurrent disease, given the relatively high incidence of GCR in recurrent disease with excellent outcome (median OS: 36 months), significantly better than incomplete resection, in the present study. Therefore, recurrent gastric cancer patients with resectable metastatic lesion(s) confirmed by complete radiologic evaluations may be the best candidates for surgical resection before palliative chemotherapy. A randomized trial is also essential to prove the benefit of GCR in both primary metastatic and recurrent disease. However, the implementation of such a study may be difficult due to several practical reasons. For example, in the present study, 62.9% of primary metastatic gastric cancer patients with palliative surgical resection had been considered to be curatively resectable cases before surgery.

The outcome of patients with palliative resection before chemotherapy seems to be better with relatively small but significant proportion of long-term survivors (5-year OS: 20.2%), compared to that of western studies also analyzing patients treated with palliative resection and chemotherapy^12,16,18^. The possible explanations for more favorable OS of surgical resection group in the present cohort are as follows. First, in Korea and Japan, surgical resection can be performed with minimal risk of postoperative complications due to highly experienced surgeons and less prevalent comorbidities of patients, allowing early initiation of palliative chemotherapy^39^. Second, Asian gastric cancer patients tend to have favorable characteristics such as relatively lower frequency of proximal lesions, liver metastasis and undifferentiated histology^39,40^. Third, second- or further- line of chemotherapy is more commonly performed in Korea and Japan^40^. For example, in the present study, almost two thirds of patients with surgical resection received second- or further- line chemotherapy. Finally, a significant proportion of GCR (41.2%) might contribute to the favorable outcome with long-term survivors, although direct comparison with other studies was difficult due to very few reports with the data of completeness of palliative resection^8^.

There were several limitations in the current study. First, it is a retrospective single institution study. Second, various chemotherapy regimens including both single and combination therapy were used. Third, patients who had not been referred to medical oncology department or had not been suitable for chemotherapy even after referral due to poor PS or morbidity after surgical resection were not included in analysis. Nonetheless, the present study compared the outcomes of patients treated with surgical resection followed by palliative chemotherapy with those of patients who underwent palliative chemotherapy alone, analyzing the entire cohort including both primary metastatic and recurrent gastric cancer patients who underwent palliative chemotherapy using third-generation agents in almost all cases in a single institution, during the specific period and with fairly mature follow-up duration (minimum follow-up duration of survivors: 31 months). Therefore, the current study may reflect the treatment results in real-world practice setting. Moreover, even after PSM, palliative surgical resection was still beneficial in terms of OS.

In conclusion, the present study suggests that judicious use of surgical resection before chemotherapy in RPMGC patients may result in a favorable outcome, especially when complete resection is achievable, although large scale phase III trials are essential to establish this treatment approach as a standard practice.
